# Molecular crosstalk between COVID-19 and Alzheimer’s disease using microarray and RNA-seq datasets: A system biology approach

**DOI:** 10.3389/fmed.2023.1151046

**Published:** 2023-06-07

**Authors:** T. Premkumar, S. Sajitha Lulu

**Affiliations:** Department of Biotechnology, School of Bio Sciences and Technology, Vellore Institute of Technology, Vellore, Tamil Nadu, India

**Keywords:** COVID-19, Alzheimer’s disease, regulatory networks, comorbidity, biomarkers

## Abstract

**Objective:**

Coronavirus disease 2019 (COVID-19) is an infectious disease caused by Severe Acute Respiratory Syndrome Coronavirus-2 (SARS-CoV-2). The clinical and epidemiological analysis reported the association between SARS-CoV-2 and neurological diseases. Among neurological diseases, Alzheimer’s disease (AD) has developed as a crucial comorbidity of SARS-CoV-2. This study aimed to understand the common transcriptional signatures between SARS-CoV-2 and AD.

**Materials and methods:**

System biology approaches were used to compare the datasets of AD and COVID-19 to identify the genetic association. For this, we have integrated three human whole transcriptomic datasets for COVID-19 and five microarray datasets for AD. We have identified differentially expressed genes for all the datasets and constructed a protein–protein interaction (PPI) network. Hub genes were identified from the PPI network, and hub genes-associated regulatory molecules (transcription factors and miRNAs) were identified for further validation.

**Results:**

A total of 9,500 differentially expressed genes (DEGs) were identified for AD and 7,000 DEGs for COVID-19. Gene ontology analysis resulted in 37 molecular functions, 79 cellular components, and 129 biological processes were found to be commonly enriched in AD and COVID-19. We identified 26 hub genes which includes *AKT1, ALB, BDNF, CD4, CDH1, DLG4, EGF, EGFR, FN1, GAPDH, INS, ITGB1, ACTB, SRC, TP53, CDC42, RUNX2, HSPA8, PSMD2, GFAP, VAMP2, MAPK8, CAV1, GNB1, RBX1*, and *ITGA2B*. Specific miRNA targets associated with Alzheimer’s disease and COVID-19 were identified through miRNA target prediction. In addition, we found hub genes-transcription factor and hub genes-drugs interaction. We also performed pathway analysis for the hub genes and found that several cell signaling pathways are enriched, such as PI3K-AKT, Neurotrophin, Rap1, Ras, and JAK–STAT.

**Conclusion:**

Our results suggest that the identified hub genes could be diagnostic biomarkers and potential therapeutic drug targets for COVID-19 patients with AD comorbidity.

## 1. Introduction

SARS-CoV-2 (Severe Acute Respiratory Syndrome-Corona Virus Disease 2019) become a major health issue and highest prevalence rate (1). According to the world health organization (WHO) report worldwide, the COVID-19 outbreak affected over 600 million people and 6.8 million of them died, as of 6 march 2023 a total of 1.3B vaccine doses have been administrated.^1^ SARS-CoV-2 genome consists of 29,811 nucleotides of enveloped positive-stranded ssRNA; as a result, SARS-CoV-2 appears to bind exclusively to angiotensin-converting enzyme 2 (ACE2) (2). This causes severe acute respiratory distress. ACE2 expression levels are highest in the small intestine, testis, heart, kidneys, and thyroid and the lowest in the brain, bone marrow, spleen, blood, blood vessels, and muscle (3). COVID-19 vaccines were developed and deployed rapidly, successfully controlled the pandemic, and reduced the risk of associated death and severe illness (4–6). COVID-19 poses a greater risk of death for patients with pre-existing neurological conditions (7). Virus RNA transcripts and viral proteins were also found in brain tissues of COVID-19 patients during an autopsy (8, 9). Neurological symptoms have been reported in COVID-19 cases more notably in recovered patients from COVID-19 challenged memory loss and cognitive disability (10). Clinical studies have proven the possibility of COVID-19 pathogenesis in the brain, and, some studies pointed out that COVID-19 might accelerate the neurodegeneration of Alzheimer’s Disease (AD) and Parkinson’s Disease (5, 11–15). As a result of COVID-19, cognitive impairment may be caused by the following mechanisms like Direct COVID-19 infection in CNS, Systematic hyperinflammatory response to COVID-19, Peripheral organ dysfunction, Severe coagulopathy, Cerebrovascular ischemia due to endothelial dysfunction, and Mechanical ventilation due to severe disease conditions (16, 17).

Alzheimer’s Disease is a neurodegenerative disorder more than 50 m people are affected worldwide and this count is expected 150 m in 2050 (18). The major reason for AD is a breakdown of amyloid precursor protein (APP) in the brain which generates beta-amyloid (Aβ) in extracellular neural space (19–21). Several enzymes reported for the breakdown of APP importantly three secretase enzymes such as alpha-secretase, beta-secretase, and gamma-secretase play crucial roles in the cleavage process (22–24). Another possible mechanism of AD is an intracellular hyperphosphorylated tau protein (25). The tau protein plays a vital role in the stabilization and assembly of microtubules, as well as in regulating plasticity and synaptic function. Tau protein hyper phosphorylates under certain physiological conditions, resulting in the destabilization of associated microtubules, synaptic damage, and other complications (26, 27). A higher permeability of BBB might permit viruses and bacteria to enter the brain (28). Several pathogens are implicated in the development of AD, including viruses, bacteria, fungi, and parasites (29). COVID-19 crosses the BBB and induces an inflammatory response within microvascular endothelial cells leading to BBB dysfunction (16, 30). In previous studies, integrated bioinformatics and system biology approaches also investigated the impact of SARS-CoV-2 on neurological disease progression (31–33). Systems biology provides a comprehensive interpretation of high-throughput platforms including genomics, proteomics, and metabolomics for analysis, display, compatibility, and accessibility. Comorbidity analysis for diverse diseases has become possible with the availability of high-throughput data and system biology bioinformatics approaches also provides a better way to unravel the biological complexity of these multifactorial diseases influenced by multiple pathogenic determinants (34, 35). To investigate the molecular factors that influence the development of SARS-CoV-2 and neurological comorbidities, we investigated multiple gene expression datasets from AD and SARS-CoV-2 which includes microarray data and transcriptome data from various human brain tissue and blood samples. We proposed a network-based systems biology approach to explore the relationship between AD and SARS-CoV-2.

## 2. Materials and methods

### 2.1. Data collection

We have used gene expression datasets such as transcriptome datasets and microarray datasets to find the differentially expressed genes. This collection of datasets was extracted from gene expression omnibus (GEO) at the National Center for Biotechnology Information^2^ (36, 37).

For our analysis, we used the following inclusion criteria:

1. Dataset which contains samples from the disease group and the control group in original experimental studies.
2. Expression profiling by array used for AD with GEO2R tool support.
3. Expression profiling by high throughput sequencing with raw counts data used for COVID-19.
4. Only homo-sapiens datasets were included.
5. A dataset containing at least eight samples included.

The keywords used for AD include “Alzheimer’s Disease” and further the results were filtered by the term “homo-sapiens,” and we selected the study type “expression profiling by array” which resulted in five datasets for AD. Among the five datasets, three of them were associated with peripheral blood mononuclear cells (PBMCs), and two of them were brain tissue-based. For COVID-19 we used the keywords “SARS-CoV-2” to narrow down the results and further filtered them by “homo-sapiens,” and “expression profiling by high-throughput sequencing.” We retrieved three datasets related to COVID-19, including two PBMC datasets and one brain tissue dataset. Both control (non-diseased) and diseased samples are included in all the datasets Table 1.

**Table 1:** Microarray datasets obtained from the GEO database with the search key terms “Alzheimer’s Disease” and “SARS-CoV-2” with a filter restricting to “Homo Sapiens.”

S. No	Accession ID	Platform	Sample count (case/control)	Analysis methods
1	GSE4226	GPL1211, NIA MGC, Mammalian Genome Collection	AD;14/14	GEO2R
2	GSE4229	GPL1211, NIA MGC, Mammalian Genome Collection	AD;12/28	GEO2R
3	GSE18309	GPL570, Affymetrix Human Genome U133 Plus 2.0 Array	AD;6/3	GEO2R
4	GSE97760	GPL16699, Agilent-039494 Sure Print G3 Human GE v2 8x60K Microarray	AD;9/10	GEO2R
5	GSE36980	GPL6244, Affymetrix Human Gene 1.0 ST Array	AD;33/47	GEO2R
6	GSE152418	GPL24676, Illumina NovaSeq 6,000	COVID;17/17	DESeq2
7	GSE166190	GPL20301, Illumina HiSeq 4,000	COVID;11/11	DESeq2
8	GSE174745	GPL24676, Illumina NovaSeq 6,000	COVID;6/3	DESeq2

Expression type microarray and RNA-Seq to Alzheimer’s Disease and SARS-CoV-2, respectively.

### 2.2. Preprocessing and identification of differentially expressed genes

To classify genes with significantly different expression levels between samples, differential gene expression analysis is necessary. GEO2R was used to identify DEGs from microarray data, the selected microarray datasets have two groups control and disease (37, 38). The (Linear Models for Microarray Data) limma Bioconductor package is also available in GEO2R online tool for finding the differentially expressed genes (39). As part of the normalization process, outliers were removed using the log2 transform, and the Benjamin Hackenberg methods are used by default to correct *p* value (40). To perform DEGs analysis, we selected false discovery rate (FDR) *p* values adjusted for multiple testing. We downloaded the full table with the following columns for further analysis value of *p*, adjusted value of *p*, log fold change, gene symbol, and title (41). Following DEGs, we plotted a volcano plot using the pheatmap package in R, genes with *p* value <0.05, and log FC | > 1 was considered (42).

For transcriptomics datasets, we have used a DESeq2 Bioconductor package (version 3.16) in RStudio version 2022. The transcriptome profile of COVID-19 tissues and blood samples was compared with control tissues and blood samples. DESeq2 is a statistical model designed to identify differentially expressed genes between two or more conditions, it is often used in the analysis of RNA-Seq data, to identify the genes which change in expression between different biological samples or conditions (43, 44). The DESeq2 model uses a negative binomial distribution to model the count data obtained from RNA-Seq experiments and variance for each gene across all samples. The model accounts for technical variability such as differences in sequencing depth, and for biological variabilities such as differences in cell size or the presence of outliers (44).

Once the mean and variance for each gene are estimated, the DESeq2 model uses a hypothesis testing framework to determine which genes are significantly differentially expressed between the conditions of interest. The resulting *p* value and log fold changes are then used to rank the genes based on their level of differential expression (45, 46).

### 2.3. Identification of common gene ontology terms and identification of overlapped genes among COVID-19 and Alzheimer’s disease

Followed by preprocessing and DEGs identification of COVID-19 and AD datasets, we classified them into four different groups AD-PBMC, AD-Tissue, COVID-19-PBMC, and COVID-19 -Tissue (47). To identify the overlapped gene among these four groups, a Venn diagram was created using an online Venn diagram tool Interactive Venn.^3^ Then the identified common genes were taken for constructing a (Module 1) PPI network for further analysis. Web-based database for annotation visualization and integrated discovery (DAVID)^4^ tool was used to perform a gene ontology analysis for DEGs for Alzheimer’s disease and COVID-19 independently (48). We have taken only those genes with common GO terms among AD and COVID-19 for further analysis and constructed a PPI network (Module 2).

### 2.4. Protein–protein interaction analysis and hub genes prediction

The biological functions and possible associations are mainly carried out by the PPI and we constructed two PPI networks. The first protein interaction network (Module 1) was constructed using the common differentially expressed genes between the four groups and on other hand, the PPI network (module 2) was constructed using the genes with common GO terms. The protein interactions were constructed using STRING version 11.5^5^ online tool then the PPI network was analyzed and visualized through Cytoscape software^6^ (49). The protein interaction networks are large networks and every node is connected with an edge, the highly interconnected genes (edges) in the PPI network consider hub genes. After constructing the two PPI networks we used the CytoHubba plugin version 0.1 in Cytoscape to identify the highly connected genes (50). Four topological features or ranking methods such as maximal clique centrality (MCC), Degree, Closeness, and Betweenness were employed to identify the hub genes. We have collected the top 20 genes from every method, and the gene present in at least three ranking methods were considered hub genes (51).

### 2.5. Analysis of transcription factor and microRNAs of hub genes

The interaction between hub genes-transcription factors (TFs) and hub genes-microRNAs (miRNA) has been conducted. Transcription factors play a crucial role, it binds with specific genes and regulates the rate of transcription of genetic information. Bioinformatically and/or *in vitro* assessment is possible of some of the mechanistic functions of candidate miRNAs prior to conducting preclinical animal tests (52). Cytoscape iRegulon plugin version 1.3 was used to predict the potential interactions between hub genes and TFs. In iRegulon, the enriched motifs were ranked depending on the direct targets using the position weight matrix (53). Therefore, AD and COVID-19 associated hub genes miRNA targets were predicted by using miRDB (MicroRNA Target Prediction Database).^7^ The miRNA targets predictive score (rank) >80 was considered a reliable score (54). The identified miRNAs were further plotted using Cytoscape software. For a better understanding of the role of miRNAs in disease mechanisms, we identified the hub miRNAs using four ranking methods (Degree, betweenness, closeness, and stress) of the CytoHubba plugin in Cytoscape (55, 56).

### 2.6. Drug-gene interaction analysis of hub genes

**Figure 1:**
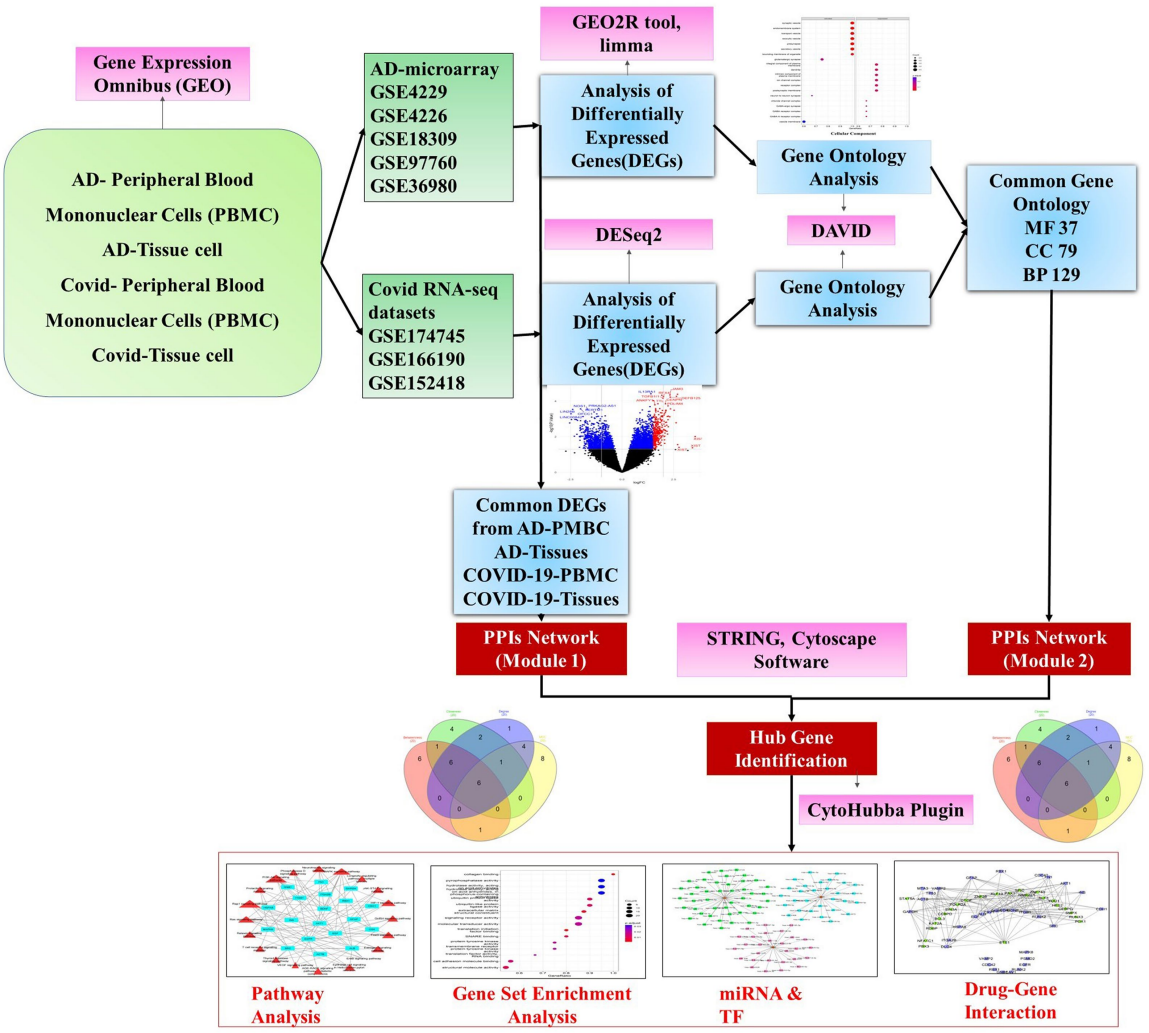
A schematic diagram of the workflow adopted in the study depicting the major steps of preprocessing of microarray and RNA-Seq data followed by identification of differentially expressed genes using R packages and gene ontology and hub gene analysis. Further, the hub genes were exposed to pathway analysis, miRNAs, and transcription factor prediction.

The drug-gene interaction was identified using Drug Gene Interaction Database (DGIdb) (57). DGIdb interface provides a search for genes against a database of drug-gene interactions and druggable targets. FDA approval status was confirmed through the drug bank database for shortlisted drugs in the interaction (Figure 1).

### 2.7. Gene ontology and pathway analysis of hub genes

Cluster Profiler (Version 4.1.0) Bioconductor package in R was used for creating Gene ontology of the hub genes (58). The top gene-ontology of molecular function (MF), cellular component (CC), and biological process (BP) were plotted using a bubble plot, and biochemical pathways associated with hub genes were identified using the KEGG database (Kyoto encyclopedia genes and genomes) (59).

## 3. Statistical analysis

### 3.1. DEGs

DEGs were identified for each data set by using adjusted *p*-values based on the moderated t-statistic (adj P) <0.05 along with an absolute value of logFC (log foldchange) of >1. The logFC ≥1 was considered as upregulated genes and logFC ≤ −1 was considered as downregulated genes.

### 3.2. Gene set enrichment analysis

The enrichment analysis of the gene ontology terms was confirmed using the “cluster Profiler” package, the analysis was performed separately for each comparison with applied hypergeometric statistical test, through the below equation,


$$P=1-\overset{k-1}{\underset{i=0}{\sum }}\frac{\left(\begin{array}{c} M\\ i \end{array}\right)\;\left(\begin{array}{c} N-M\\ n-i \end{array}\right)}{\left(\begin{array}{c} N\\ n \end{array}\right)}$$

*p*-values were adjusted for multiple comparisons, and *q*-values were also calculated for FDR control as well. *p*-values <0.05 were considered to be significantly enriched terms (58).

### 3.3. Gene ontology and pathway analysis

In DAVID, Fisher’s Exact test is adopted to measure the gene enrichment in annotation terms. Fisher’s Exact *p*-values are computed by summing probabilities P over defined sets of tables (Prob = ∑Ap). The modified Fisher exact *p*-value (EASE score) ≤ 0.05 and FDR < 0.05 are considered strongly enriched (60, 61).

### 3.4. Protein interaction network constructions

Protein interactions are assessed and integrated using the STRING database which includes direct (physical) and indirect (functional) associations. PPI networks can be constructed by calculating the distance ‘D’ between pairs of proteins (u,v),


$$D\;\left(u,v\right)=\frac{2|\mathrm{Nu}\cap Nv|}{\left|\mathrm{Nu}|+|Nv\right|}$$

STRING tool provides four thresholds as a default including low (0.15), medium (0.40), high (0.70), and highest (0.90) and, we created a network using a medium threshold value (61).

## 4. Results

### 4.1. Analysis of microarray and transcriptome datasets

**Figure 2:**
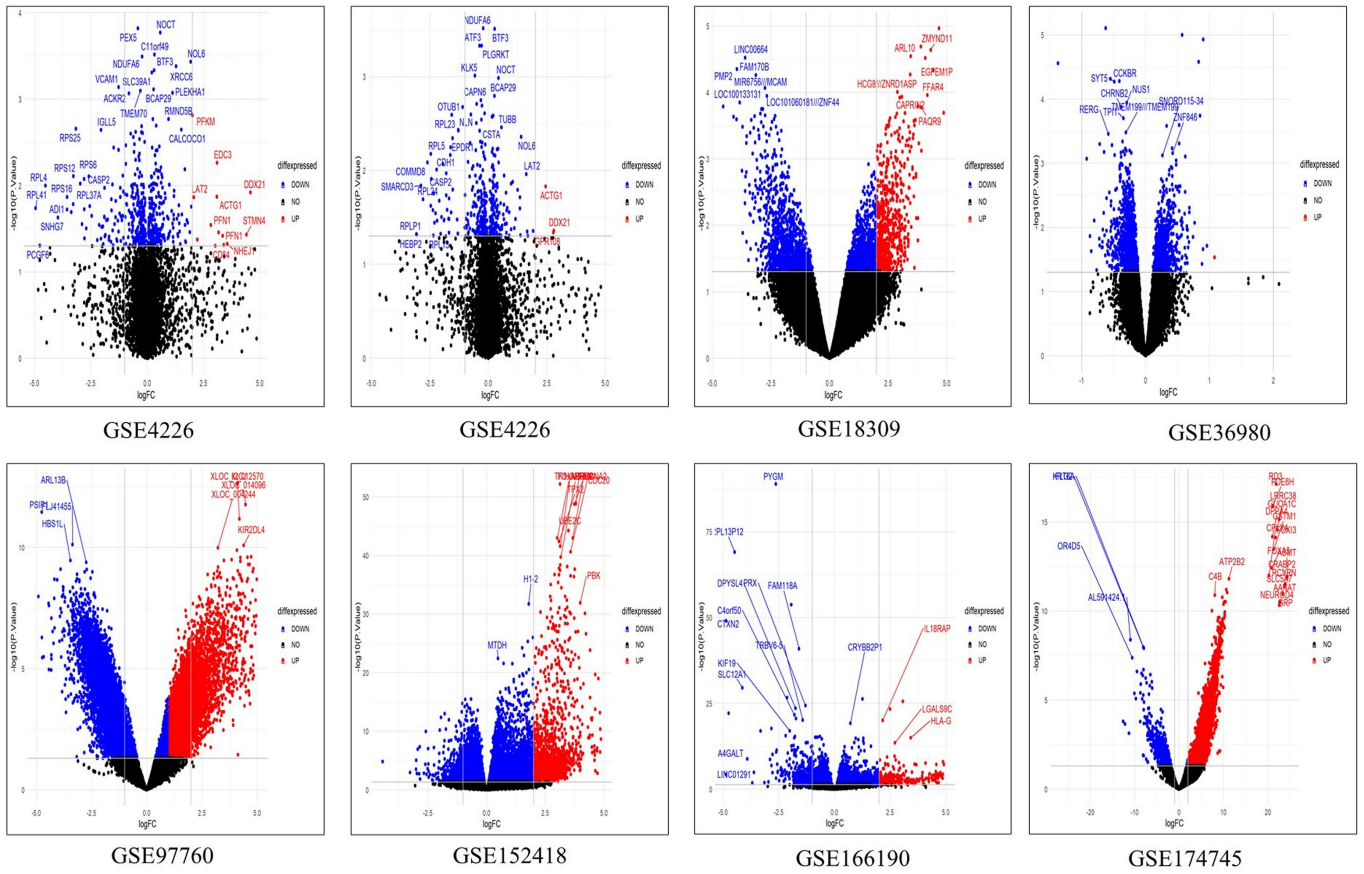
The multiple volcano plot showing differentially expressed genes of COVID-19 and AD (upregulated genes in red and downregulated genes in blue).The *x*-axis depicts the log fold change in gene expression between different samples and the *y*-axis depicts FDR-adjusted *p* values.

We retrieved five microarray datasets for AD and three transcriptome datasets for COVID-19 which includes disease and healthy samples. The AD microarray datasets were GSE4226, GSE4229, GSE18309, GSE97760, and GSE36980 analyzed through GEO2R. The transcriptome-based COVID-19 datasets GSE152418, GSE166190, and GSE174745 were analyzed through the DESeq2 Bioconductor package in R software. The datasets were analyzed individually and identified the DEGs (Supplementary Tables S1, S2). The overall upregulated and downregulated DEGs were tabulated in Table 2. Followed by DEGs the datasets were classified to four different groups such as AD-PBMC, AD-Tissue, COVID-PBMC, and COVID-Tissue in order to identify a common gene. Figure 2 demonstrates the volcano plots of the AD and SARS-CoV-2 datasets, where the red dot represents a gene that has been upregulated, and the blue dot represents a gene that has been downregulated.

**Table 2:** Differentially expressed genes of Alzheimer’s disease and COVID-19 datasets with details of upregulated and downregulated genes and total counts after deletion of duplication.

Sample groups	Datasets	Up regulated	Down regulated	Total DEGs	Duplication removed
	GSE4226	2,560	656	18,550	7,944
AD- PBMC	GSE4229	16	318		
	GSE18309	983	886		
	GSE97760	4,733	8,398		
AD-Tissue	GSE36980	1,612	1,121	2,733	1,611
COVID-19-PBMC	GSE152418	1,115	2,545	8,840	5,165
	GSE166190	206	4,974		
COVID-19 Tissue	GSE174745	1867	534	2,401	1864

### 4.2. Identification of common genes

**Figure 3:**
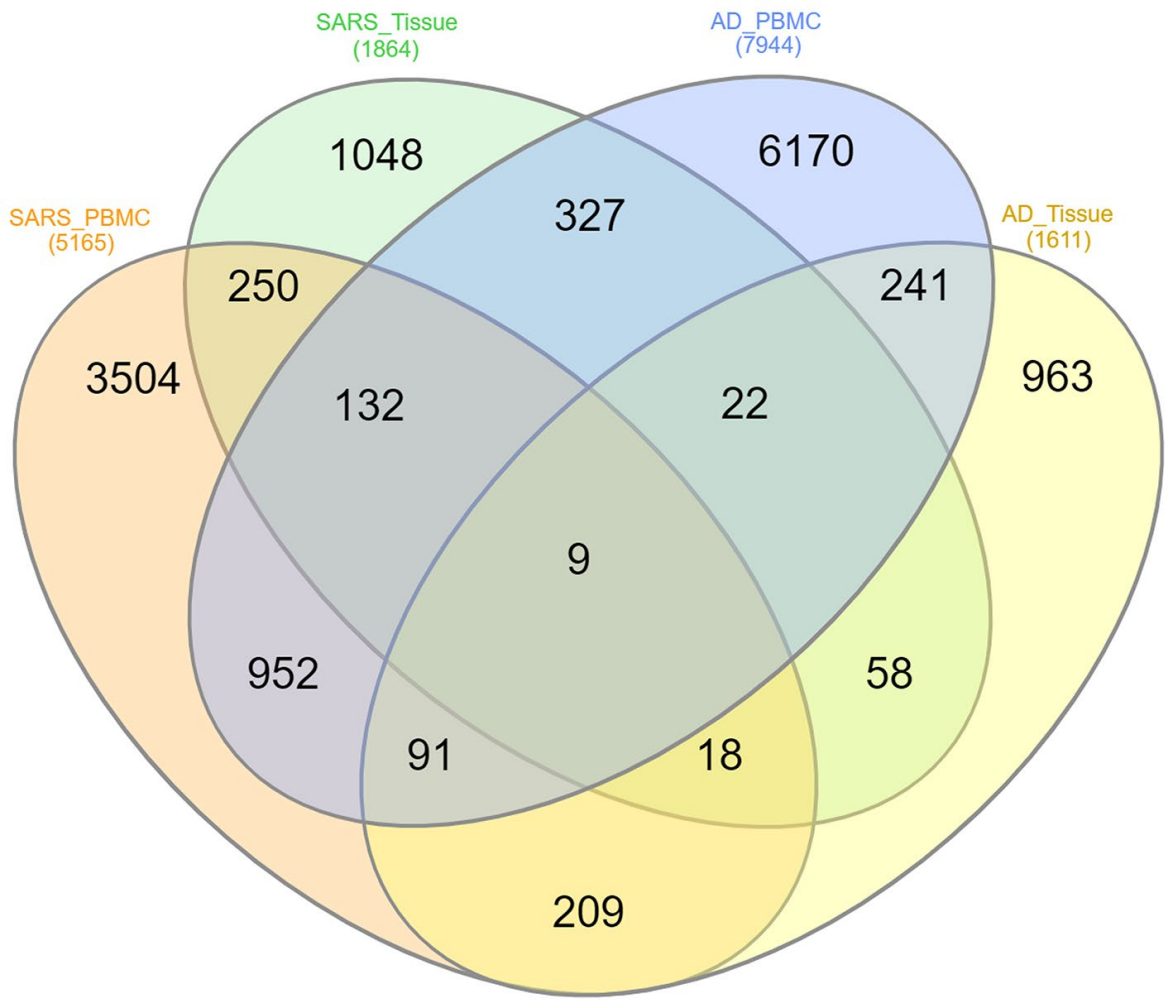
Venn diagram of shared differentially expressed genes, where each ellipse represents AD-PBMC, AD-Tissue, COVID-19-PBMC, and COVID-19-Tissue with Nine (*HST6, POLR3G, SLC6A20, ITGA2B, HOMER3, GMPR, AGBL1, CRABP2, OLFML2B*) genes common among the four groups.

The overlapped genes among the four groups are depicted in the Venn diagram Figure 3 for better understanding. Only 9 (*HST6, POLR3G, SLC6A20, ITGA2B, HOMER3, GMPR, AGBL1, CRABP2, OLFML2B*) genes have been found to be shared between AD-PBMC, AD-Tissue, COVID-19-PBMC, and COVID-19-Tissue. We identified the genes which were present in at least 3 groups and tabulated them (Table 3) for further analysis and construct a (Module 1) PPI network.

**Table 3:** Common genes identified among AD-PBMC, AD-Tissues, COVID-19-PBMC, and COVID-19-Tissues.

S. No	Datasets	Common Genes
1.	AD-PBMC, AD-Tissue, COVID-19-PBMC, COVID-19-Tissue	9
2.	AD-PBMC, COVID-19-PBMC, COVID-19-Tissue	132
3.	AD-Tissue, COVID-19-PBMC, COVID-19-Tissue	22
4	AD-PBMC, AD-Tissue, COVID-19-PBMC	327

### 4.3. Identification of common gene ontology terms among COVID-19 and Alzheimer’s disease datasets

DAVID analysis was performed to understand the biological significance of AD and COVID-19 DEGs. We found 164 MF, 175 CC, and 581 BP were enriched in Alzheimer’s disease and 146 MF, 196 CC, and 545 BP were enriched in COVID-19 datasets and 37 MF, 79CC and 129 BP were found to be commonly enriched between Alzheimer’s disease and the COVID-19 dataset. For this study, we have considered only the common GO terms for further analysis and (module 2) protein interaction network construction. Supplementary Table S4 gives the details of the commonly enriched GO terms.

### 4.4. Protein interaction network construction and analysis

**Figure 4:**
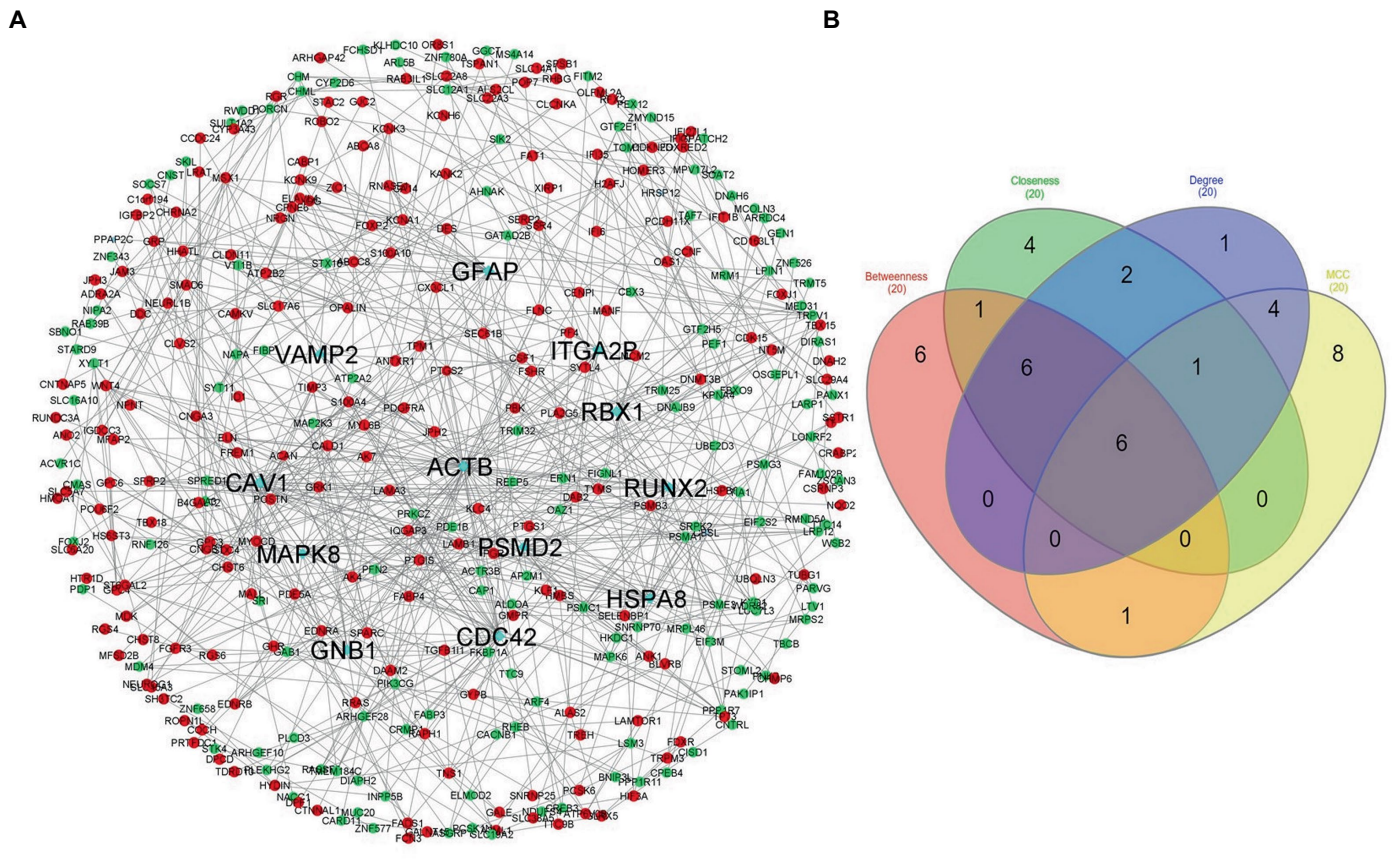
Network of protein–protein interaction and detected hub genes (from genes common among AD-PBMC, AD-Tissue, COVID-19-PBMC, and COVID-19-Tissue, module 1). **(A)** The up-regulated and down-regulated genes in red and green colors and hub genes in aqua. **(B)** Venn diagram representing the genes commonly shared among the topological features of MCC, Betweenness, Closeness, and Degree.

**Figure 5:**
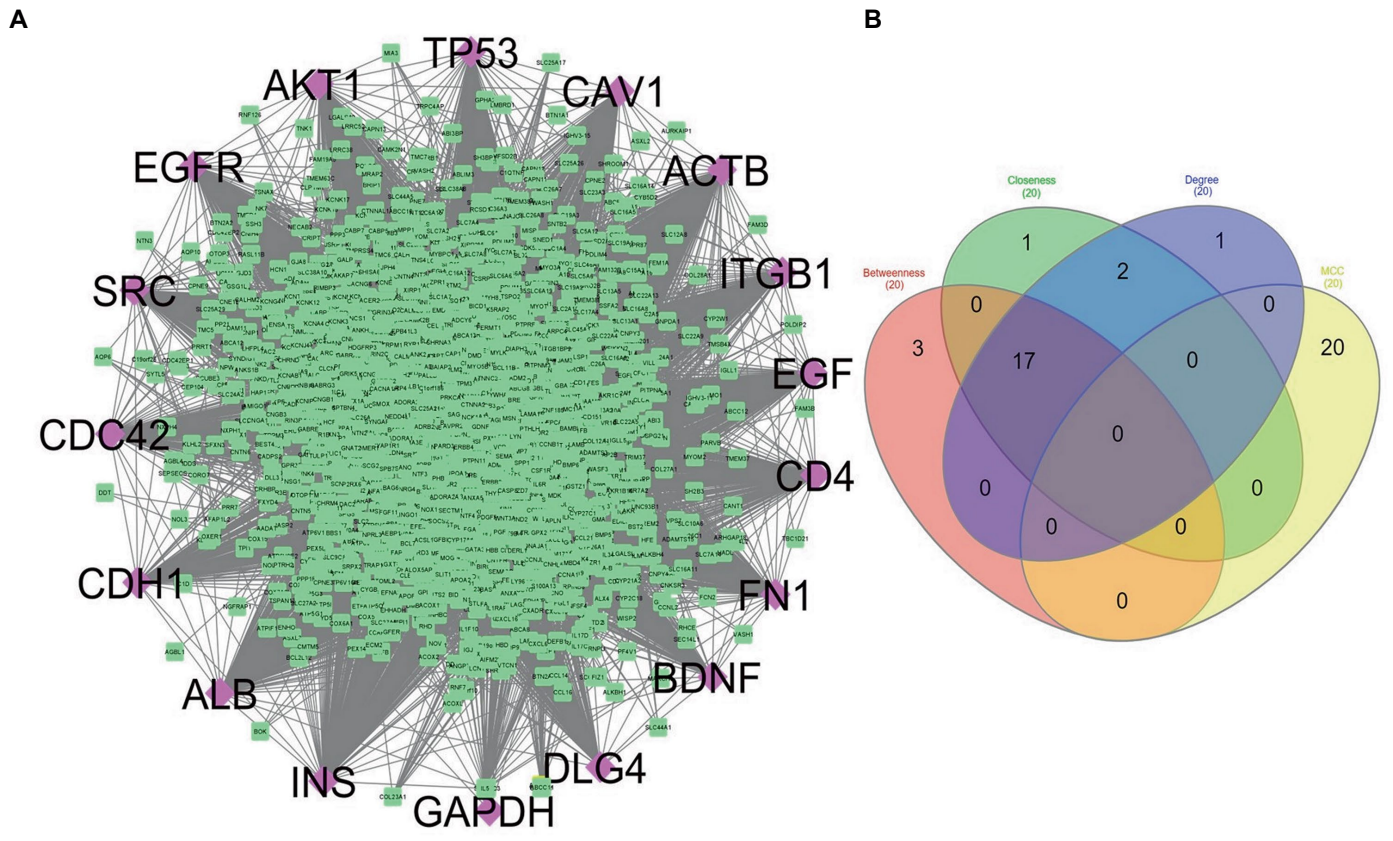
**(A)** Network constructed to represent the common genes shared by ontology terms of Alzheimer’s disease and COVID-19 gene ontology terms (module 2). Purple diamonds represent the hub genes of this network. **(B)** Venn diagram showing the genes commonly shared among the topological features of MCC, Betweenness, Closeness, and Degree.

The STRING database was used to construct the protein interaction network then visualized *via* Cytoscape software. The edges represent the interactions between the genes, and the nodes represent the genes. Figure 4 illustrates the (Module 1) PPI network of common genes with 823 edges and 373 nodes. Figure 5 illustrates the (Module 2) PPI network of GO sources with 2,674 nodes and 50,719 edges established according to the results.

### 4.5. Hub genes identification

Using the CytoHubba plugin of Cytoscape, we identified the highly interacting hub genes for the progression of AD and SARS-CoV-2. Four different algorithms, namely MCC, Degree, Betweenness, and Closeness were utilized to extract the hub genes from module 1 and module 2. We obtained the top 20 genes from both modules based on these four ranking methods and tabulated them in module 1 (Table 4) and module 2 in (Table 5). The gene present in at least 3 ranking methods are considered as hub genes. As a result, Figure 4 displays the list of hub genes (*ACTB, CDC42, RUNX2, HSPA8, PSMD2, GFAP, VAMP2, MAPK8, CAV1, GNB1, RBX1, ITGA2B*) obtained from common genes (module 1) PPI network. A group of 17 (*AKT1, ALB, BDNF, CAV1, CD4, CDC42, CDH1, DLG4, EGF, EGFR, FN1, GAPDH, INS, ITGB1, ACTB, SRC, TP53*) overlapping genes was obtained through gene ontology (module 2) PPI network (Figures 5A,B). We identified that *CAV1, CDC42*, and *ACTB* genes are common among the two sets of hub genes. The expression of Caveolin-1 (Cav-1) has been associated with aging in both senescent cells and aged tissues *in vitro* and *in vivo*. In murine embryonic fibroblasts, Cav-1 knockout accelerates premature senescence, while loss of Cav-1 accelerates neurodegeneration and aging. In most cell types, ACTB (Actin-Beta) is abundantly and stably expressed and is commonly used to normalize gene expression as an internal control (62). ACTB variant rs852423 has been found to be associated with increased susceptibility to AD (63). The identified module 1 and module 2 hub genes and their major roles are tabulated in Supplementary File 2.

**Table 4:** The top 20 genes from module 1 of (common genes of Alzheimer’s disease and COVID-19 tissues and blood) protein–protein interaction network analyzed using four different topological analysis methods such as MCC, Closeness, Betweenness, and Degree through CytoHubba plugin.

S. No	Betweenness	Closeness	Degree	MCC
1.	ACTB	ACTB	ACTB	PSMA1
2.	CDC42	CDC42	CDC42	PSMD2
3.	RUNX2	HSPA8	RUNX2	PSMC1
4.	HSPA8	RUNX2	GFAP	PSME3
5.	GFAP	CAV1	HSPA8	PSMB3
6.	ITGA2B	GFAP	CAV1	ACTB
7.	CAV1	MAPK8	GNB1	RUNX2
8.	SNRNP70	PTGS2	PSMD2	POSTN
9.	RBX1	VAMP2	MAPK8	ELN
10.	GNB1	PRKCZ	ITGA2B	SPARC
11.	VAMP2	PIK3CG	PSMA1	ACAN
12.	PIK3CG	ITGA2B	PTGS2	SPRED1
13.	MAPK8	WNT4	ACAN	TP73
14.	FKBP1A	RBX1	PSME3	TIMP3
15.	MYL6B	ACAN	RBX1	OAZ1
16.	PSMD2	PGR	TRPV1	MAPK6
17.	SLC12A1	PSMD2	PSMB3	GFAP
18.	ABCC8	GNB1	PSMC1	CDC42
19.	OAZ1	TRPV1	KCNA1	HSPA8
20.	HMBS	MAP2K3	VAMP2	SDC4

**Table 5:** The identified top 20 genes from module 2 (common gene ontology terms between Alzheimer’s disease and COVID-19) of protein–protein interaction network analyzed using four topological analysis methods such as MCC, Closeness, Betweenness, and Degree through CytoHubba plugin.

S. No	Betweenness	Closeness	Degree	MCC
1.	SRC	STAT3	STAT3	NDUFA6
2.	CFTR	DLG4	CDH1	UQCRH
3.	CAV1	CAV1	BDNF	ATP5MF
4.	ACTB	ACTB	MMP9	NDUFB7
5.	EGF	ERBB2	EGFR	NDUFV2
6.	BDNF	BDNF	ALB	ATP5PO
7.	ALB	ALB	AKT1	NDUFC2
8.	ITGB1	ITGB1	ITGB1	NDUFB6
9.	TP53	TP53	TP53	P13073
10.	INS	INS	INS	UQCRC1
11.	CDC42	CDC42	CD4	ATP5PD
12.	CYCS	EGF	DLG4	COX5A
13.	AKT1	AKT1	ACTB	NDUFB9
14.	CDH1	CDH1	CDC42	ATP5ME
15.	FN1	FN1	FN1	UQCR10
16.	SNCA	SRC	SRC	ATP5PF
17.	EGFR	ESR1	ERBB2	NDUFA12
18.	GAPDH	GAPDH	GAPDH	NDUFA8
19.	DLG4	EGFR	EGF	NDUFV1
20.	CD4	CD4	CAV1	ATP5MG

### 4.6. MicroRNAs network of hub genes

**Figure 6:**
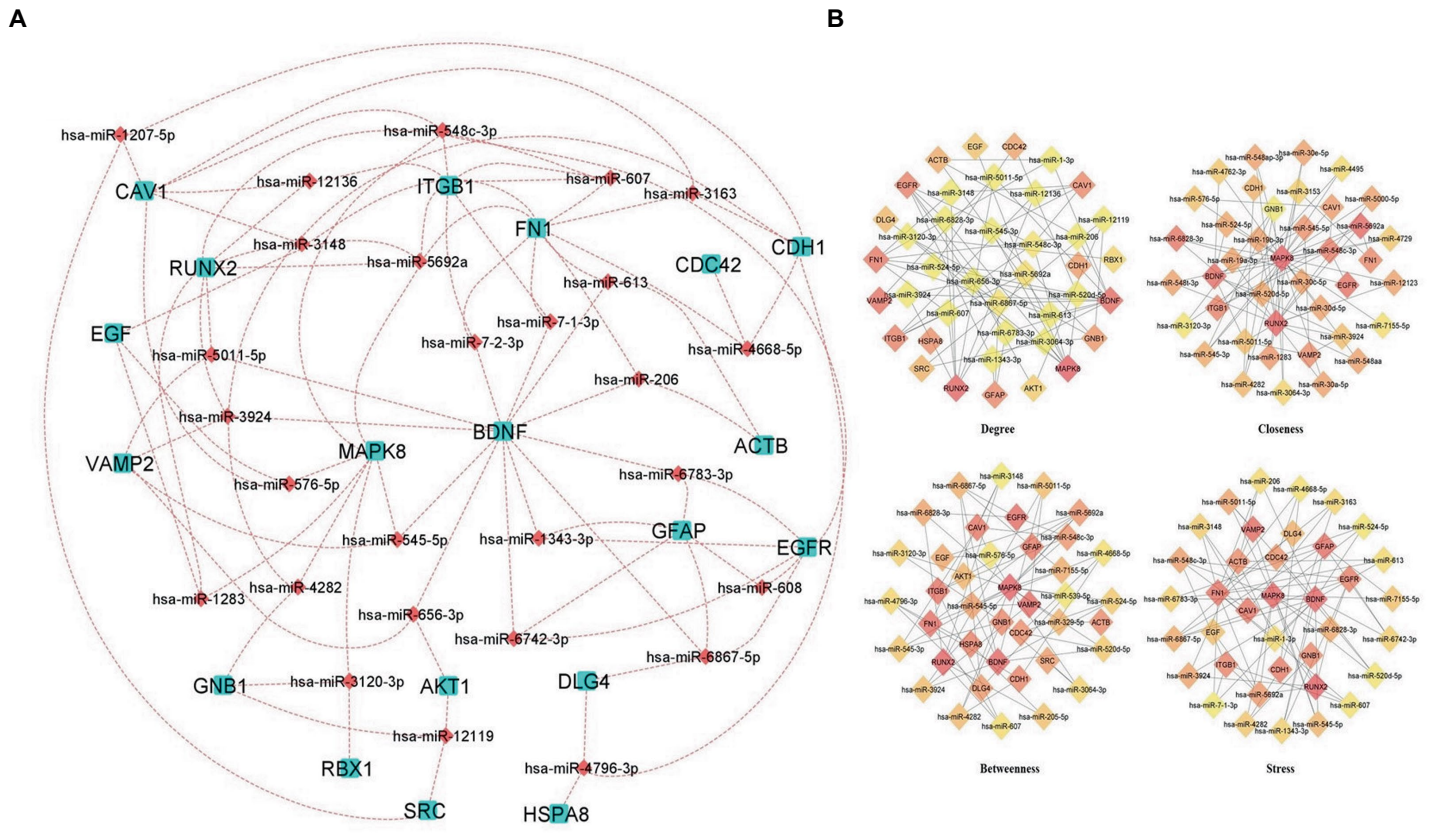
Hub Genes-miRNAs Network **(A)** miRNAs interacting with more than three hub genes, aqua color squares representing the hub genes and the maroon color diamonds representing the miRNAs. **(B)** Predicted hub miRNAs using four topological features of CytoHubba including Betweenness, Closeness, Degree, and Stress.

The regulatory networks such as miRNAs and TFs of the hub genes were identified. MicroRNAs (miRNA) and transcription factors (TFs) are involved in the development and progression of COVID-19 and its comorbid conditions. Based on the analysis of the hub genes-miRNA and hub genes-Transcription factors, we have obtained a clear network of interactions. The results revealed that the miRNAs regulate 26 hub genes, which could be a possible target of the comorbidity. All the hub genes have targeted a total of 839 miRNAs of which 27 miRNAs were targeted in more than three hub genes (Figure 6A; Supplementary Table S5).

Also, we have identified the hub miRNAs using four ranking methods (Degree, betweenness, closeness, and stress) of the CytoHubba plugin in Cytoscape. We extracted the top 40 nodes from each ranking method and the overlapped miRNAs were identified using a Venn diagram (Figure 6B; Supplementary Table S6). The miRNAs present at least three ranking methods considered as hub-miRNAs and we found five hub-miRNAs including hsa-miR-6,867-5p, hsa-miR-548c-3p, hsa-miR-6,828-3p, hsa-miR-545-5p, and hsa-miR-5,011-5p.

### 4.7. Transcription factor network of hub genes

**Figure 7:**
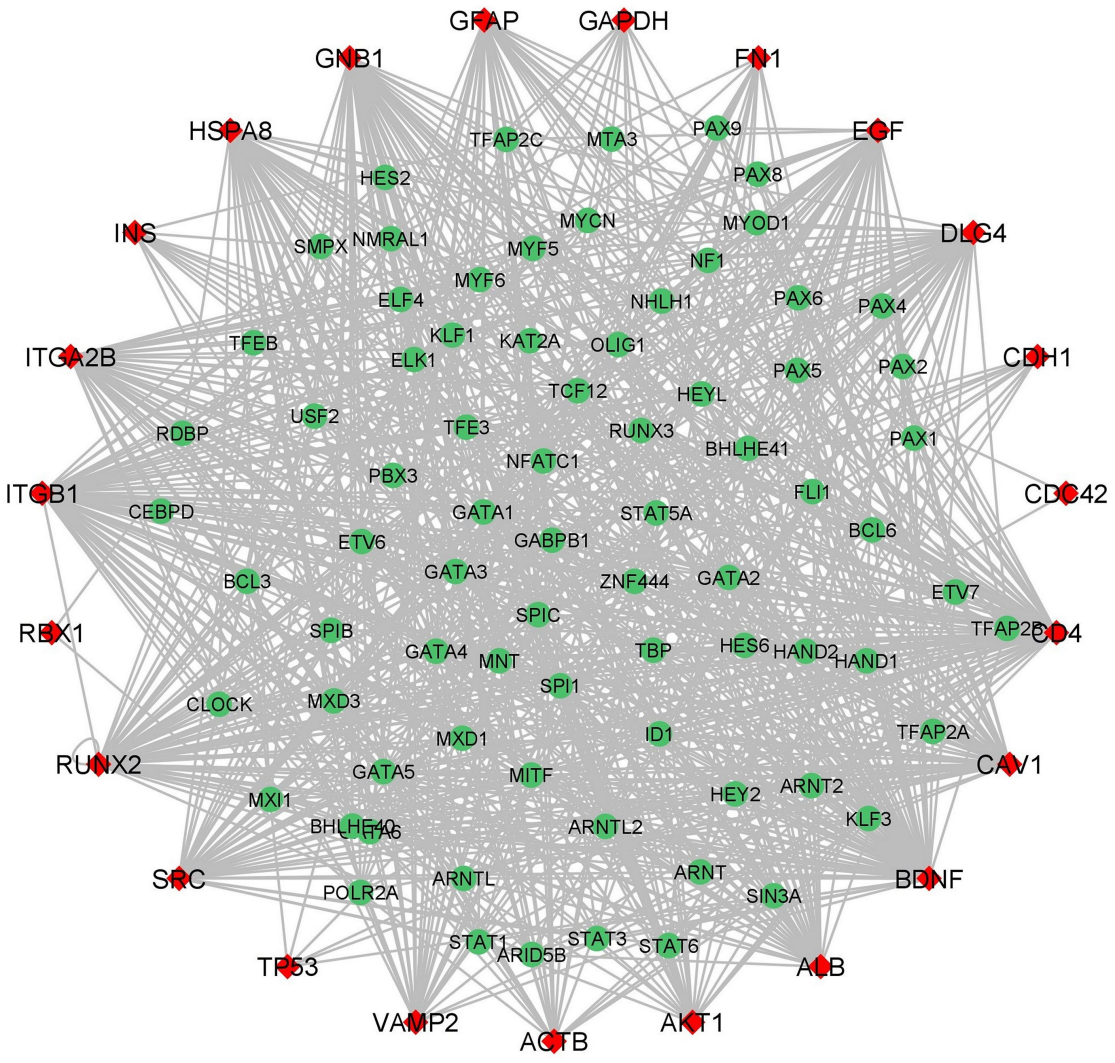
Hub Genes-Transcription Factors network (red color diamond designates the hub genes and the green color circulars designate the Transcription Factors). The edges between the two genes indicates the interaction between TFs and hub genes.

iRegulon predicted 85 TFs for the hub genes and importantly four TFs HAND2, GATA1, GATA2, and GATA6 interacted with 23 hub genes (Figure 7; Supplementary Table S7). The heart-and neural crest derivatives expressed protein-2 (HAND2) play a crucial role in neural crest development (64). The synergistic activation between HAND2 and GATA4 TFs is causally linked to congenital heart diseases (CHD). Severe CDH may contribute to delayed brain development, thromboembolism, and pulmonary hypertension. The transcription factors might play a major role in different cell types. GATA family TFs are zinc finger DNA binding proteins, GATA1 and GATA2 play an essential role in developing and maintaining the hematopoietic system (65). Jin Chu et al. reported that GATA1 acts as a transcription repressor for gamma-secretase activating protein (gsap) gene expression (66). Interestingly previous studies suggested that GATA1 is a transcription repressor for synapse-related genes. In neurological conditions such as AD, NGB may have therapeutic and disease-preventing properties that can be explored experimentally (67).

### 4.8. Identification of drug-gene interaction

**Figure 8:**
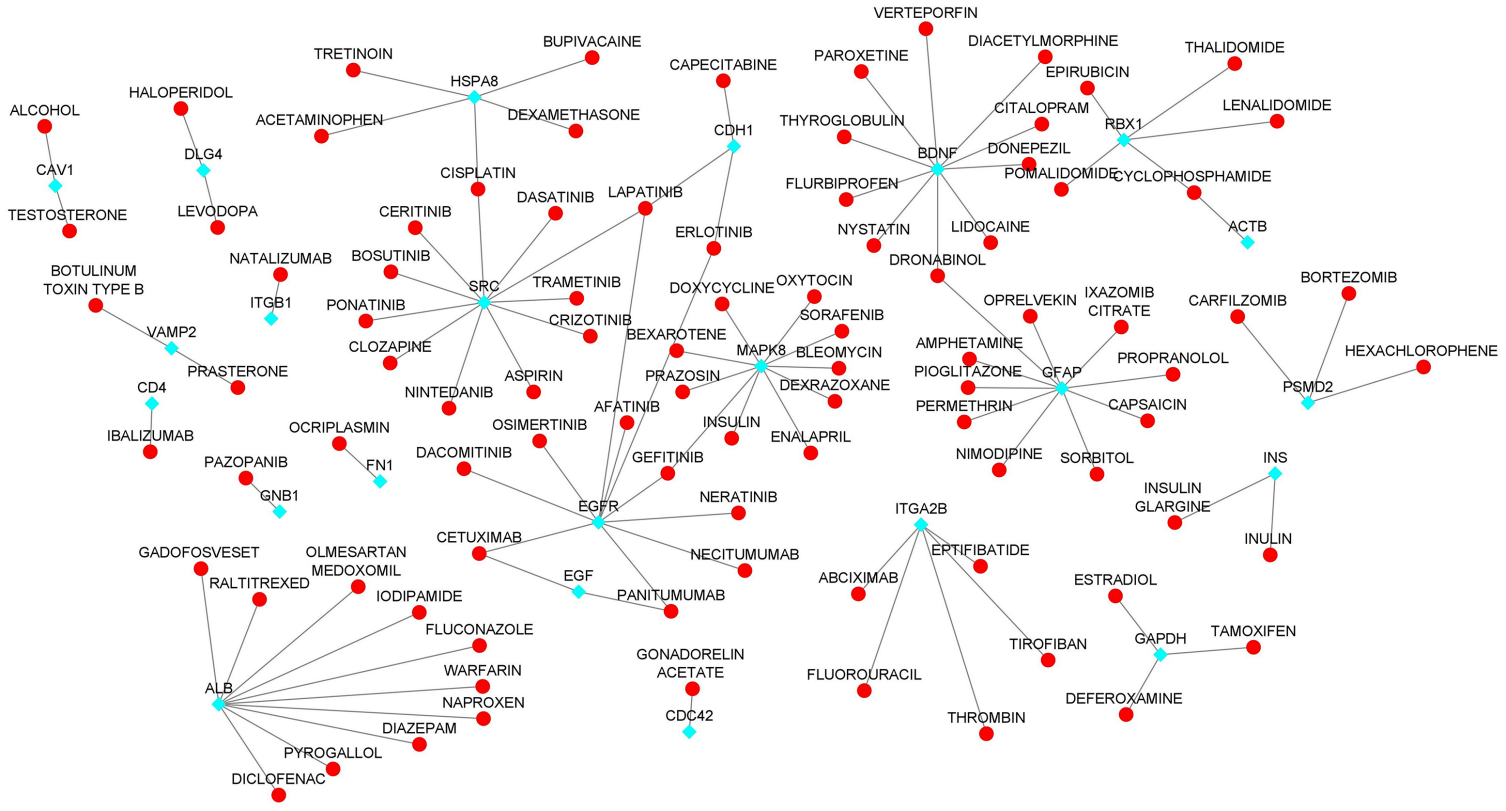
Drug-Hub Gene Network (aqua color indicating the hub genes and red color indicating the drugs).

We investigated the drug interactions of hub genes using the DGIdb. A total of 26 hub genes were explored through the drug-gene interactions network. The network result shows that a total of 106 were interacting with the hub genes (Figure 8; Supplementary Table S8). Some of the drugs were already approved by the food and drug administration (FDA) which makes this drug more possible to treat AD and COVID-19 comorbidity. There are potential therapeutics for COVID-19 comorbidities associated with the dysregulation of the proteins.

### 4.9. Gene set enrichment analysis of hub genes

**Figure 9:**
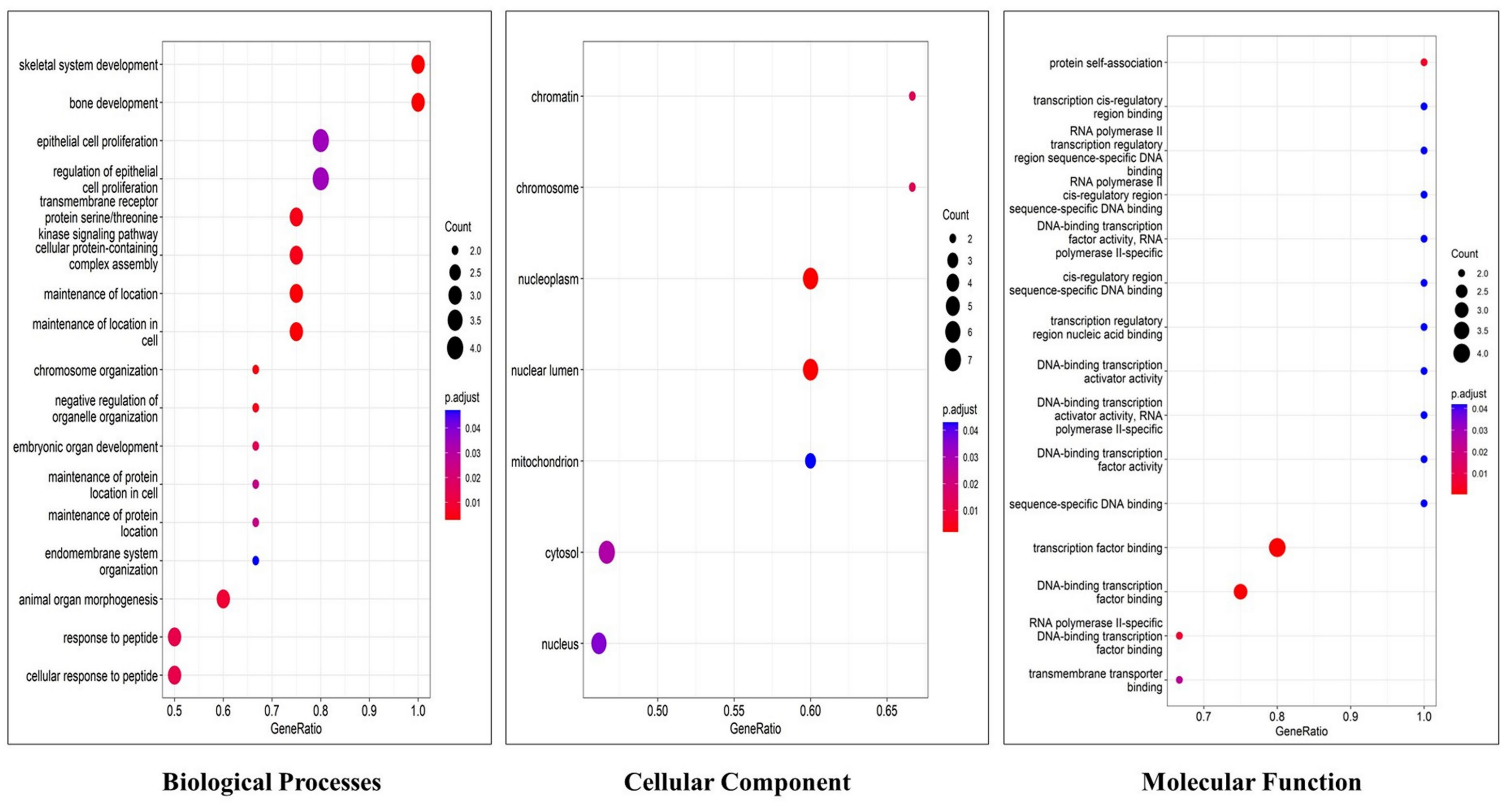
Top 20 gene ontology terms of hub genes (The *x*-axis label represents the gene ratio and the *y*-axis label represents gene ontology terms).

**Figure 10:**
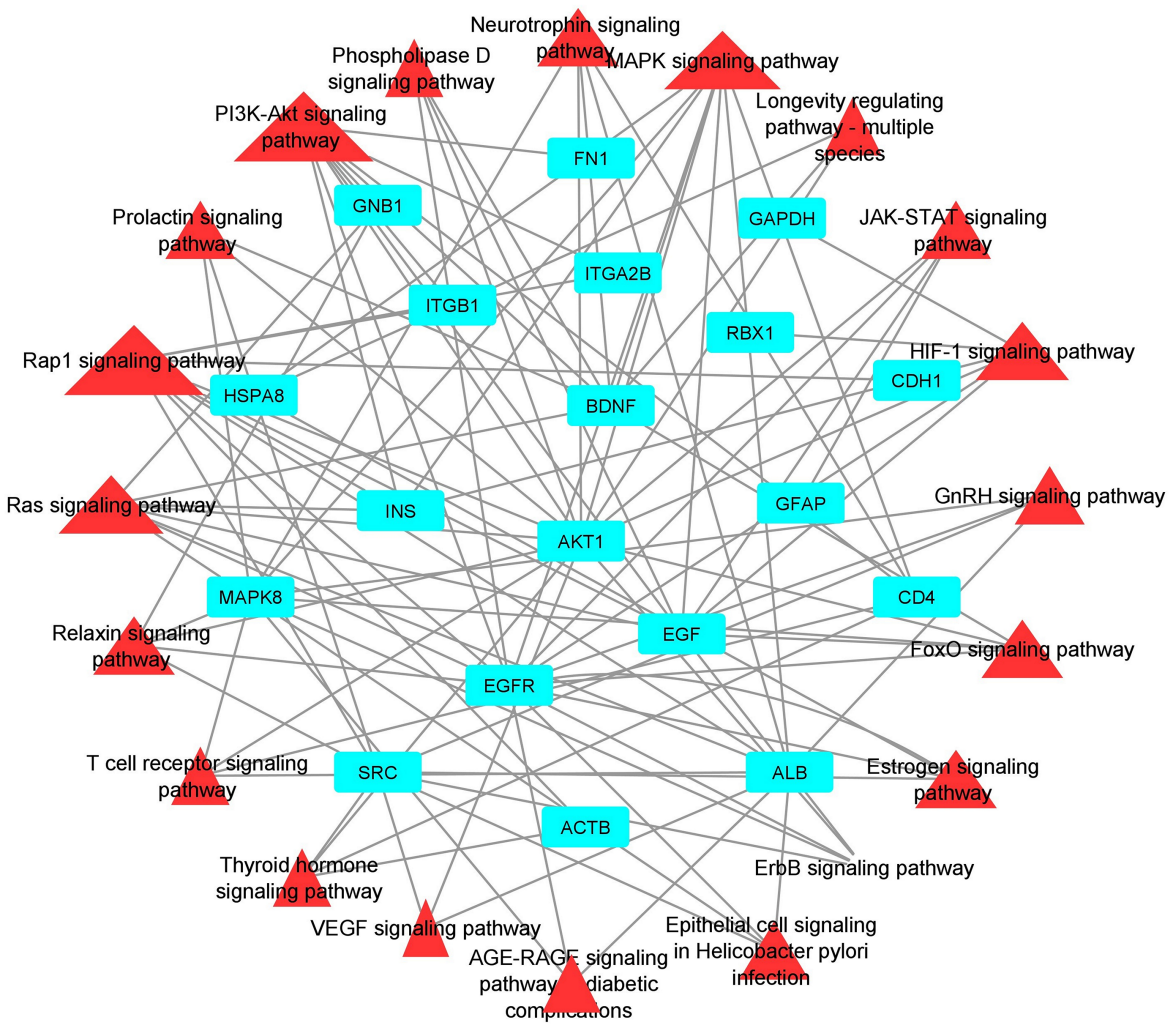
Pathway-Hub Gene Network (aqua color indicating the hub genes and the red color indicating the signal pathways).

Functional enrichment analysis results showed that hub genes are involved in several biological functions. We identified hub genes related gene ontology using cluster profiler package in r, and we plotted the significantly enriched terms based on adjusted *p* value <0.05, as illustrated in Figure 9. There are several pathways were enriched in KEGG analysis including the PI3K-AKT, Neurotrophin, Rap1, Ras, and JAK–STAT signaling pathways, and the top 20 signaling pathways are depicted in Figure 10 (Supplementary Table S9). The gene set enrichment results clearly show that the hub genes are majorly involved in the signaling pathways which might be closely linked to COVID-19 and AD.

## 5. Discussion

High-throughput sequencing technologies, bioinformatics, and systems biology analysis methods could identify and reveals the changes in the expression level of genes and also assists to identify the potential biomarkers for several diseases importantly neurodegenerative diseases. In this study, the focus is on understanding how AD and COVID-19 disease are related through pathogenetic processes and molecular crosstalks. We followed systems biology approaches including DEGs identification, PPI network construction, hub genes identification, gene set enrichment analysis, and pathway analysis. Also, we explored and identified the regulatory network and drug-genes interaction of the hub genes. To investigate the relationship between AD and COVID-19 we performed gene set enrichment analysis using AD and COVID-19 DEGs discretely. The datasets were further classified into four different groups such as AD-PBMC, AD-Tissue, COVID-19-PBMC, and COVID-19-Tissue. We collected the common DEGs from among the four groups for constructing a Protein–Protein interaction network (module 1). While only 9 DEGs (*HST6, POLR3G, SLC6A20, ITGA2B, HOMER3, GMPR, AGBL1, CRABP2,* and *OLFML2B*) were commonly expressed between these groups. In addition, we performed Gene Set Enrichment Analysis for the DEGs of Alzheimer’s disease and SARS-CoV-2 DEGs, then we retrieved the genes with common gene ontology terms for constructing a PPI network (module 2).

The *HST6, ITGA2B, HOMER3*, and *CRABP2* genes have not been reported in AD or COVID-19 related articles. In the extracellular matrix, Olfactomedin Like 2B (OLFML2B) is the olfactomedin domain protein photomedin-2, with an important role in neural crest development and neurogenesis, cell–cell adhesion, and cell cycle regulation. The OLFML2B gene may contribute to the treatment of bladder cancer in the future based on individual prognostic markers (68). Hongde Liu proposed that GMPR’s (Guanosine Monophosphate Reductase) GMPR1 is associated with Tau phosphorylation in AD *via* the AMPK (AMP-activated protein kinase) and adenosine receptor pathways (69). A therapeutic strategy of inhibiting GMPR1 with lumacaftor has been proposed to treat AD based on the elevated expression of GMPR in this disease. Wei Dong et al. explored the common initiative molecular pathways in AD and ischemic stroke and they found that AGBL1 is a common risk gene (70). SLC6A20 appears to be a novel regulator of glycine and proline levels in the brain according to the research of Mihyun Bae. Further, pharmacologically inhibiting SLC6A20 may contribute to the treatment of brain disorders *via* an increase in glycine levels in the brain and N-Methyl-D-Aspartate receptors (NMDAR) activity (71). Some important biological processes, including spliceosome genes, were dysregulated by POLR3B genes. A number of transcription factors, including FOXC2 and GATA1, play a role in neuronal dysfunction and intellectual disability, which are affected by impaired protein synthesis and splicing (72).

miRNAs as biomarkers: miRNA subsets have shown clinical relevance as biomarkers according to a growing number of reports. There are emerging miRNA therapeutics that are used to determine the presence of pathology, as well as the progression, genetic links, and stage of the disease. miRNAs have been translated into clinical medicine faster than ever because of the bioinformatic approach to identifying miRNA-binding sites and their related biological pathways in target genes, as well as the expanding availability of *in vitro* and *in vivo* preclinical research models (73). The miRNA helps to understand the development and progression of COVID-19 and AD comorbidity. In the miRNAs network *BDNF, MAPK8, ITGB1, FN1, EGFR*, and *RUNX2* hub genes are associated with most of the miRNAs. The co-expression network revealed that hsa-miR-6,867-5P regulates EGFR, DLG4, GFAP, BDNF and hsa-miR-548C-3p regulates EGFR, MAPK8, ITGB1, CAV1 and hsa-miR-5692a regulates ITGB1, FN1, MAPK8, EGF, RUNX2. Research suggested that hsa-miR6867-5P and 6,867-5P were associated with platelet apoptosis and adhesion in an autoimmune disease like immune thrombocytopenia (74). Recent studies exhibited that hypothalamic miRNAs including miR-548C-3p are potential contributors to different neurodegenerative diseases, also this author identified 29 novel hypothalamic MicroRNAs as a propitious therapeutic regimen for SARS-CoV-2 by regulating ACE2 and TMPRSS2 expression (75). Cosin et al. studied a multiple linear regression model for predicting amyloid beta levels in Cerebrospinal fluid, for this they used four validated miRNAs for AD including miR-545-5p, miR-142-3p, miR-34a-5p, and miR-15b-5p. The results revealed that miR-34a-5p is the best-predicting miRNA for amyloid beta levels in cerebrospinal fluid (Cosín-Tomás et al., 2017). The miR-545-3p, and miR-34a-5p could be potential biomarkers for the early detection of AD (Cosín-Tomás et al., 2017).

To illustrate the mechanisms of hub genes we performed enrichment analysis including GO and pathway analysis. We found various cell signaling pathways are enriched including RAP1, MAPK, PI3K-AKT, RAS, and HIF-1 signaling pathways, etc. The signaling pathway of RAP1 was found to be a crucial regulator of cellular functions such as the formation and control of cell adhesion and junction and, also plays a major role during cell invasion and metastasis in different cancers (76). MAPK pathway responds to numerous extracellular stimulations including inflammatory cytokines, stress, and viral infection. Furthermore, COVID-19 infection activated MAPK and the downstream signaling possibly leading to cell death. Intense work is in progress to develop a compound to target MAPK pathways to treat neurodegenerative and inflammatory diseases (77). Proliferation, apoptosis, and angiogenesis, the Renin-angiotensin signaling pathway (RAS) has been shown to play a role in tumorigenesis through complex interactions (78). Krishna Sriram et al. reported that RAS has a great tendency to cause comorbidities and mortality and they proposed a model to predict effective drugs to target RAS (79). RAS–ERK signaling induces amyloid precursor protein and tau protein hyperphosphorylation which are enhanced in AD brains, and inhibition of RAS-MAPK activation prevents tau and amyloid precursor protein hyperphosphorylation (80). HIF-1α (hypoxia-inducible factor) plays a crucial role in inflammatory responses, regulating metabolic pathways and regulating the aging process. Dysregulations of the pathway HIF-1α lead to several diseases including cardiovascular disease, cancer, and AD. HIF-1α is a key activator for COVID-19 and inflammatory responses and it could be a therapeutic target for virus-induced inflammatory diseases and COVID-19 (81). As part of the immune response and virus entry into the cell, Phosphatidylinositol 3-kinase (PI3K)/AKT signaling plays a significant role also this pathway is involved in several aspects of neurological disease development (82). Patients with COVID-19 have been found to have an increased risk of lung tissue fibrosis following activation of the PI3K-AKT signaling pathway (83). Cancers and diabetes are associated with excessive activation of the PI3K-AKT pathway also cardiovascular diseases and neurological conditions such as AD and PD might also be affected by the deregulation of the pathway (84). Enriched BP of hub genes has primarily participated in the cellular response to peptides, animal organ morphogenesis, endomembrane system organization, maintenance of protein location, and embryonic organ development. The top enriched terms of CC were nucleus, cytosol, mitochondrion, nuclear lumen, and nucleoplasm. The top five terms in MF were mainly enriched transmembrane transporter binding, RNA polymerase II-specific DNA-binding transcription factor binding, DNA binding transcription factor binding, sequence-specific DNA binding and transcription factor binding. We constructed a drug-gene network for hub genes and investigated the relationship between the chemical and the disease. Through this drug-gene network, we found several drugs including diacetylmorphine, donepezil, dronabinol, levodopa, haloperi, deferoxamine, raltitrexed, diazepam, and warfarin. These drugs are already reported for treating AD and Parkinson’s disease (85–89). Recent studies reported repurposing of CNS drugs are potential to treat SARS-CoV-2-infected individuals (90). We have found an interaction between DEGs-miRNAs-TFs which are plays key roles in the pathogenesis of neurological disorders.

It is necessary to acknowledge that the study has some limitations because it only relies on bioinformatics and network biology. One of the limitations of the study is the potential confounding effects associated with the variations in transcriptome profiles from different tissues (brain vs. blood). Also selecting overlapping DEGs from separate analyses of tissues and blood samples may not completely eliminate the confounding effect of sample variation. Additionally, the large number of DEGs identified in the study may have caused a potential for false positive results. While we attempted to address these issues by performing additional analyses including hub genes and pathway analysis.

## 6. Conclusion

The present study aims to understand the molecular crosstalk between COVID-19 and Alzheimer’s Disease, including discovering the gene expression signatures, TFs, Drug-gene interaction, miRNAs associations, and dysregulated molecular pathways. As a result of integrated analyses of microarrays and transcriptomics of PBMC cells and tissue cells, we were able to identify AD and COVID-19 DEGs. Through PPI network analysis twenty-three (*AKT1, ALB, BDNF, CAV1, CD4, CDC42, CDH1, DLG4, EGF, EGFR, FN1, GAPDH, INS, ITGB1, ACTB, SRC, TP53, RUNX2, HSPA8, PSMD2, GFAP, VAMP2, MAPK8, GNB1, RBX1, ITGA2B*) hub genes were identified. Transcription factor network analyses revealed that several TFs play a crucial role in post-transcriptional and transcriptional regulators of the differentially expressed genes. The identified shared pathways between AD and COVID-19 provide there are several similar underlying mechanisms play in both diseases. Our findings could lead to identifying a potential biomarker to predict the highest risk of neurological complications with COVID-19. Also, the identified transcription factor might be a potential therapeutic drug target for both diseases.

## Data availability statement

The datasets presented in this study can be found in online repositories. The names of the repository/repositories and accession number(s) can be found in the article/Supplementary material.

## Author contributions

TP analyzed the data and wrote the manuscript. SS conceptualized and designed the work, revised, and edited the manuscript. All authors contributed to the article and approved the submitted version.

## Conflict of interest

The authors declare that the research was conducted in the absence of any commercial or financial relationships that could be construed as a potential conflict of interest.

## Publisher’s note

All claims expressed in this article are solely those of the authors and do not necessarily represent those of their affiliated organizations, or those of the publisher, the editors and the reviewers. Any product that may be evaluated in this article, or claim that may be made by its manufacturer, is not guaranteed or endorsed by the publisher.

## Footnotes

^1^ https://covid19.who.int/

^2^ https://www.ncbi.nlm.nih.gov/

^3^ http://www.interactivenn.net/

^4^ https://david.ncifcrf.gov/

^5^ https://string-db.org/

^6^ https://cytoscape.org/

^7^ https://mirdb.org/
