# Supplementary material for: Molecular crosstalk between COVID-19 and Alzheimer’s disease using microarray and RNA-seq datasets: A system biology approach
Source: Front Med (Lausanne). 2023 Jun 7;10:1151046. doi: 10.3389/fmed.2023.1151046 (PMC10286240; doi:10.3389/fmed.2023.1151046)
Supplement: Supplementary file 2 [file Table_2.docx]

**Supplementary File 2:** Identified hub genes from modules 1 and 2 and their important roles in biological function

| **S. No** | **Gene symbol** | **Full name** | **Function** | **References**  **(PMID)** |
| --- | --- | --- | --- | --- |
|  | ACTB | Actin Beta | During the process of polymerization of actin, filaments form cross-linked networks within a cell cytoplasm | 29581253 |
|  | AKT1 | AKT Serine/Threone Kinase 1 | During adult neurogenesis, AKT regulates the tempo of the integration of newborn neurons, including correct neuron positioning, dendritic development, and synapse formation, through the AKT-mTOR signaling pathway | 15526160, 11882383 |
|  | ALB | Albumin | Its main function is the regulation of the colloidal osmotic pressure of blood | 19021548 |
|  | BDNF | Brain-Derived Neurotrophic Factor | Participates in axonal growth, pathfinding, and dendritic growth and morphology modulation | 11152678 |
|  | CAV1 | Caveolin 1 | Decreased caveolin-1 (Cav-1) expression, a membrane/lipid raft (MLR) scaffolding protein necessary for synaptic and neuroplasticity | 33981778 |
|  | CD4 | CD4 Molecule | plays an essential role in the immune response and serves multiple functions in responses against both external and internal offenses | 34798897 |
|  | CDC42 | Cell Division Cycle 42 | CDC42 is required for the formation and maintenance of filopodia, thin projections of surface actin-rich strands in neurons | 14978216 |
|  | CDH1 | Cadherin 1 | CDH1 is involved in mechanisms regulating cell-cell adhesions, mobility and proliferation of epithelial cells | 11976333 |
|  | DLG4 | Discs Large MAGUK Scaffold Protein 4 | Postsynaptic scaffolding protein that plays a critical role in synaptogenesis and synaptic plasticity by providing a platform for the postsynaptic clustering of crucial synaptic proteins. Required for synaptic plasticity associated with NMDA receptor signaling | 26334723 |
|  | EGF | Epidermal Growth Factor | Cell culture experiments have shown that EGFR stimulates epidermal and epithelial growth in vivo and in vitro as well as some fibroblast growth. | 10964941 |
|  | EGFR | Epidermal Growth Factor Receptor | Signaling cascades involved in converting extracellular cues into appropriate cellular responses by binding to receptor tyrosine kinases of the EGF family | 2790960, 10805725 |
|  | FN1 | Fibronectin 1 | Fibronectin plays an important role in cellular adhesion, motility, opsonization, wound healing, and the maintenance of cell shape | 3024962, 3900070 |
|  | GAPDH | Glyceraldehyde-3-Phosphate Dehydrogenase | Participates in nuclear events including transcription, RNA transport, DNA replication and apoptosis | 3170585, 11724794 |
|  | GFAP | Glial Fibrillary Acidic Protein | It appears that GFAP gene activation and GFAP protein production play a significant role in the activation of astroglia cells (astrogliosis) following CNS injuries and neurodegeneration | 25975510 |
|  | GNB1 | G Protein Subunit Beta 1 | In various transmembrane signaling systems, guanine nucleotide-binding proteins (G proteins) act as modulators or transducers | 30559307 |
|  | HSPA8 | Heat Shock Protein Family A (Hsp70) Member 8 | This gene plays an important role in protein quality control, ensuring that proteins are folded correctly, refolded when they are misfolded, and targeted for degradation after folding | 21150129, 21148293 |
|  | INS | Insulin | Cells become more permeable to monosaccharides, amino acids, and fatty acids when they are exposed to insulin | 32730766 |
|  | ITGA2B | Integrin Subunit Alpha 2b | This receptor plays a crucial role in the blood coagulation system, by mediating platelet aggregation | 26252081 |
|  | ITGB1 | Integrin Subunit Beta 1 | As a result of integrating alpha-3/beta-1 at invadopodia plasma membranes, FAP (seprase) may be involved in adhesion, engaging cell invasion, matrix degradation and formation of invadopodia | 18635536 |
|  | MAPK8 | Mitogen-Activated Protein Kinase 8 | Mapk8 regulates a variety of cellular activities including proliferation, differentiation, survival, and death | 18307971 |
|  | PSMD2 | Proteasome 26S Subunit Ubiquitin Receptor, Non-ATPase 2 | PSMD2 belongs to the 26S proteasome, which degrades ubiquitinated proteins using ATP | 32476569 |
|  | RBX1 | Ring-Box 1 | It is involved in the progression of the cell cycle, signal transduction, transcription, and nucleotide excision repair when transcription is coupled with nucleotide excision | 10230407, 10579999 |
|  | RUNX2 | RUNX Family Transcription Factor 2 | Osteoblast differentiation and skeletal morphogenesis are regulated by RUNX2 transcription factors | 28505335 |
|  | SRC | SRC Proto-Oncogene, Non-Receptor Tyrosine Kinase | Several biological activities are controlled by SRC, such as cell adhesion, immune response, gene transcription, migration, cell cycle progression, cell death, and transformation. | 7853507 |
|  | TP53 | Tumor Protein P53 | Several tumor types are suppressed by this gene; it induces apoptosis or growth arrest depending on the cell type and physiological conditions | 11025664, 12524540 |
|  | VAMP2 | Vesicle Associated Membrane Protein 2 | An important SNARE protein involved in synaptic vesicle fusion that releases neurotransmitters | 30929742 |
